# NEURON: Enabling Autonomicity in Wireless Sensor Networks

**DOI:** 10.3390/s100505233

**Published:** 2010-05-25

**Authors:** Anastasios Zafeiropoulos, Panagiotis Gouvas, Athanassios Liakopoulos, Gregoris Mentzas, Nikolas Mitrou

**Affiliations:** 1 National Technical University of Athens, Heroon Polytexneiou, 15773, Zografou, Greece; E-Mails: pgouvas@mail.ntua.gr (P.G.); gmentzas@mail.ntua.gr (G.M.); mitrou@cs.ntua.gr (N.M.); 2 Greek Research and Technology Network, Av. Mesogion 56, 11527, Athens, Greece; E-Mail: aliako@grnet.gr

**Keywords:** wireless sensor network, autonomicity, energy efficiency, clustering, hierarchical routing, p2p, overlay, NEURON

## Abstract

Future Wireless Sensor Networks (WSNs) will be ubiquitous, large-scale networks interconnected with the existing IP infrastructure. Autonomic functionalities have to be designed in order to reduce the complexity of their operation and management, and support the dissemination of knowledge within a WSN. In this paper a novel protocol for energy efficient deployment, clustering and routing in WSNs is proposed that focuses on the incorporation of autonomic functionalities in the existing approaches. The design of the protocol facilitates the design of innovative applications and services that are based on overlay topologies created through cooperation among the sensor nodes.

## 1. Introduction

In the last decade, there has been a great evolution in the sensor networking world. A vast amount of small, inexpensive, energy-efficient, and reliable sensors with wireless networking capabilities is available worldwide, increasing the number of sensor network deployments [1,2]. The adoption of IPv6, combined with the advanced networking capabilities of modern sensor nodes, enables the integration of sensor networks into the existing IP networking infrastructure [3]. IPv6 provides a huge address space for networking purposes, while concurrently leading to the rapid development of many useful applications. Following these advancements, the vision of a World-Wide Sensor Web [4] is becoming a reality, since the trend in next generation networks is to create fully interconnected infrastructures, consisting of many heterogeneous networks.

The transition to large scale wireless sensor networks (WSNs) increases the complexity in their operation and management and raises stability and scalability issues [5]. This is due to the special requirements that are imposed from the sensor nodes and the characteristics of the WSNs. Battery limitations and the need for lifetime maximization make necessary the overall power consumption minimization during operation. Energy-efficient operation has to be realized taking in account the existence of heterogeneous sensor nodes with diverse computing and storage capabilities. Furthermore, the highly volatile and dynamic nature of WSN topologies—due to continuous nodes joins and leaves or mobility in the WSN field—requires the continuous intervention of the network administrator.

Self-organization mechanisms are proposed in order to reduce the complexity in the operation and management of WSNs. Considering the specific characteristics of such networks, the management of sensor infrastructures should be as autonomic as possible, *i.e.*, the wireless sensors should manage themselves with minimum or no human intervention [6]. Autonomic functionalities have to be developed towards the sensor network evolution focusing on the introduction of functionality that will enable the provision of advanced services with low power consumption. Establishment of cooperation among nodes, ability to retrieve the current status of each sensor node, ubiquitous access to data and optimisation of the overall sensor network performance are examples of functionalities that facilitate the efficient operation of sensor nodes from the energy and performance perspective. By developing self-functionalities, autonomic monitoring will be realized, adaption to environmental changes will be supported and operational and management cost will be reduced.

A promising method to enable autonomicity in wireless heterogeneous environments is the adoption of decentralized schemes [5,7]. Centralized components make the system vulnerable in the sense that they are single points of failure. Furthermore the entire traffic load may be needed to be directed towards them, which is energy consuming, especially in dense networks [8,9]. On the contrary, decentralized schemes are fully scalable and achieve homogeneous load distribution. Current research efforts show that decentralization may be achieved through the exploitation of techniques that are applied in peer-to-peer (p2p) networks since relevant protocols rely on decentralized structures, such as Distributed Hash Tables (DHTs) [10]. These techniques are based on the creation of an overlay topology and the implementation of specific mechanisms over it [4]. P2p overlays may be formulated over sensor networks to eliminate the need for proxy support, to provide efficient data lookup, to limit broadcasts and to enable flexible access to the sensed data. However, the maintenance of an overlay topology is challenging in dynamic environments where nodes join or leave the network [11,12] and requires the exchange of a large number of messages among the participating nodes [13].

The transition to an overlay approach for the WSN deployment, operation and management has to be combined with energy efficient techniques, such as clustering and hierarchical routing techniques [14]. Nodes that are elected to operate as Cluster Heads (CHs) are involved mainly in routing and packet relaying functions while the rest nodes perform a minimum set of operations [15]. Furthermore, the available routing information may be exploited from upper layer mechanisms and make them more efficient.

In this paper we propose NEURON, a novel protocol for autonomic deployment, cluster formulation and hierarchical routing in wireless sensor networks. NEURON combines the concepts of self-organization, decentralization and optimization in building networking infrastructure. The combination of these concepts is crucial, since it permits autonomic deployment and re-configuration of the network and supports the development of self-optimisation functionalities. Energy efficiency is achieved and network lifetime is extended through clustering and hierarchical routing mechanisms. NEURON does not make any assumptions for the location of the sensors, their capabilities and the roles that may undertake. It is designed in order to be stable and scalable and to facilitate the creation of overlay topologies in the sensor network by exploiting routing information stored in the nodes.

The paper is organized as follows. Section two briefly presents the related work on the field while section three describes in detail the proposed protocol and its mechanisms. Section four details how NEURON may facilitate the autonomic provision of advanced services in a WSN. Section five discuses the simulation results and section six concludes the paper with a short summary of our work and a presentation of open research issues and future work.

## 2. Background—Related Work

### 2.1. Autonomic Characteristics and IP Integration

Since a common technology is beneficial, the trend in the design and implementation of future networks is the integration of sensors with the existing IP networks. WSNs are usually considered as low-power, wireless personal area networks (LoWPANs). LoWPANs are poised to form the next tier of the Internet by connecting billion of sensor nodes to the Internet and using the existing computing infrastructure [3,16]. Extending IP to LoWPANs was once considered impractical [3] due to the resource-intensive overhead imposed by IP communication to energy-constrained devices. In order to handle these issues, 6LoWPAN was introduced as an adaptation layer that enables efficient IPv6 communication over 802.15.4 links [3].

The transition to IP is crucial since it enables the design and deployment of autonomic functionalities in WSNs. IP networking characteristics may be exploited by the network layer in order to be responsive and adaptive while remaining energy-efficient. The deployment of autonomic functionalities is helpful since the protocols and the techniques that are being developed for monitoring and management of WSNs should have minimal configuration, preferably work “out of the box”, be easy to bootstrap, and enable the network to self heal given the inherent unreliable characteristic of these devices. The basic autonomic functionalities that have to be supported by sensor nodes are [17]:

- *Self-configuration*: change configuration parameters (e.g., data acquisition rate) according to the conditions in the sensor environment,
- *Self-optimisation*: fine-tune each sensor entity in order to achieve pre-determined goals, while low energy consumption and optimal quality of service have to be guaranteed based on corrective actions,
- *Self-healing*: detect system malfunctions or failures and start corrective actions based on defined policies and cooperation among neighbouring nodes to recover the network or a node,
- *Self-protection*: recognize possible sources of problems or threats in the sensor network and take proactive measures,
- *Self-awareness*: sense network changes in the sensor environment and be aware of the capabilities and the status of your neighbours.

Self-organizing systems completely rely on localized decision processes and nodes have to follow three basic methods in order to implement the desired behaviour; interact with other nodes in their neighbourhood, adapt their local state according to the conditions in their environment, and include probabilistic techniques in their decisions [5,18]. Based on the acquired knowledge, it is desirable that the initial sensor network configuration takes advantage of the underlying physical sensing and topological characteristics so as to assign responsibilities to nodes that are best suited to perform certain sensor network duties [19]. This preliminary assignment of duties facilitates future reorganization of the network in order to easily adapt to any changes.

Taking into account these considerations, NEURON’s mechanisms are designed to operate in a fully autonomic manner. The network entities cooperate and exchange information based on the policies that are applied by these mechanisms. Thus, the sensor network may be self-configured and self-optimised, it supports self-awareness through proper dissemination of information and is able to react to possible failures.

### 2.2. Clustering and Routing Algorithms for WSNs

Since energy efficiency is a crucial characteristic for the network lifetime in WSNs, it is necessary to design mechanisms that are not energy consuming. Several techniques have been proposed recently for energy efficient clustering and routing in WSNs. It has been proved that by using hierarchical (tiered) architectures, the network lifetime can be extended significantly [20,21]. A hierarchy may be created by using clustering mechanisms and hierarchical routing has to be selected.

Clustering has been proven to be energy-efficient since data routing and relaying are only operated by cluster heads (CHs) [22]. CHs process, filter and aggregate data sent by cluster members, thus reducing network load and bandwidth utilisation. Besides, non-CH sensors are not involved in routing and relaying data and transmissions are only operated by CHs [15]. During the clustering process, it is necessary to take into account aspects such as the cluster size and formation, criteria for CHs selection, how to control inter-cluster and intra-cluster collisions and energy saving issues [15]. For the proper design of a clustering mechanism, multiple challenges have to be addressed. Clustering has to be efficient in terms of processing complexity and message exchanges and the clustering formulation technique has to be autonomic, i.e. each node takes decisions independently of the other nodes. The clustering process has to be completed within a bounded number of iterations and, after the clusters have been formulated, each node operates either as a CH or as a simple node. Furthermore, adequate distribution of CHs over the sensor field has to be accomplished [4]. Finally, since CHs consume more energy in aggregating and routing data, it is essential to have an energy-efficient mechanism for CHs election and rotation [15].

Hierarchical routing protocols have to be correlated with the clustering formulation mechanisms in order to reduce the routing messages exchanged and, thus, decrease energy consumption [14]. These protocols are closely related to the clustering mechanism and usually support multi-hop communication, where each cluster member forwards its data to the CH and the CHs forwards the data to the gateways in the network. Since many of the proposed systems involve large networks, it is essential to provide routing infrastructures that concurrently offer small routing state and robustness [23,24]. Limiting routing states stored in the nodes is crucial for scalability and efficiency [25] while robustness entails handling efficiently topology and connectivity changes due to node failures and environmental impact. Moreover, it is important to provide mechanisms that are able to handle heterogeneous nodes with diverse capabilities in the network [26].

Energy-efficient clustering and routing algorithms for wireless sensor networks are presented in the literature [27–29]. Cluster formation is typically based on the energy reserve of sensors and sensor’s proximity to the CH [30]. Low-Energy Adaptive Clustering Hierarchy (LEACH) [15] was one of the first clustering algorithms proposed for sensor networks. LEACH is a distributed, proactive, dynamic algorithm that forms clusters of sensors based on the received signal strength and uses local CHs as routers to the sink. It includes randomized rotation of the high-energy cluster-head position, *i.e.*, delegate cluster-head functionality among the various sensors in order to preserve the battery of a single sensor. LEACH clustering terminates in a constant number of iterations but it does not guarantee good CH distribution and assumes uniform energy consumption for CHs. On the contrary, HEED [31] makes no assumptions on energy consumption and achieves to select well-distributed CHs. However, it prerequisites that the nodes in the network are quasi-stationary and have equal capabilities. EDAC [32] is an improvement of LEACH for heterogeneous environments that supports energy-driven CHs rotation and extends the node lifetimes, while LENO [33] proposes a dynamic CH rotation algorithm that outperforms both LEACH and EDAC. PEGASIS [34], LBCS [9] and EECS [35] are various enhancements of LEACH, where energy dissipation is balanced among sensor nodes under special conditions or assumptions. PEGASIS and LBCS assume that all nodes have location information about all other nodes, while EECS supposes that all nodes are stationary and uniformly dispersed within a sensor field. In all the above cases, the sensors are directly connected to their CHs. Furthermore, layered approaches exist such as SOHS [36] and EECF [15] in which sensors are organized into clusters creating a hierarchical topology and HMPR [37] where the WSN is initially constructed as a layered network. These protocols necessitate the existence of fixed centralized nodes or fixed lifetimes and identifiers for the sensor nodes. Finally, TinyHop [38] is proposed as a reactive routing protocol that aims to minimize the number of messages necessary to perform routing. Therefore, this protocol avoids the energy consuming periodic beacon messages generated by other proactive routing protocols. TinyHop may also be configured to limit the flooding of messages within the scope of the cluster. In our approach, the routing among the CHs is designed in a reactive manner, taking into consideration existing reactive protocols that are already designed for mobile *ad hoc* networks [39] such as DSR [40].

The NEURON protocol is designed as a new approach for providing autonomic and energy efficient clustering and routing in WSNs without assuming specific pre-conditions for the nodes functionality and their knowledge. As described in detail in Section 3, NEURON does not make any assumptions about the location of the sensors, their heterogeneity, their capabilities, the pre-definition of CHs or centralized nodes and their unique identifiers. This is achieved since the clustering and routing mechanisms are self-organized and are based on the awareness of the neighbouring environment of each node (*self-awareness*). Furthermore, in order to maximise network lifetime, the self-organization phase is designed in order to be short and energy efficient, characteristic that is not adequately addressed from the existing protocols [27].

### 2.3. Overlay Networking in WSNs and DHTs

Autonomic functionalities in sensor networks may be facilitated by the creation of virtual overlay topologies [41]. The creation of p2p overlay topologies was proposed in order to treat the underlying heterogeneous WSNs as a single unified network, in which interacting sensors can exchange information (store and retrieve data) without considering the details of the infrastructure underneath. P2p protocols are selected since they rely on decentralized algorithms, such as Distributed Hash Tables (DHTs). P2p overlays over (traditional) sensor networks eliminate the need for centralized proxies and provide easy publication and search for the sensed data [4]. They provide efficient data lookup, guarantees on lookup times and location independence. The overhead of building applications is distributed amongst participating nodes with no central point of failures and without global broadcasts [10,42,43].

Several approaches have been proposed for p2p overlay networking in sensor networks. In [44], a DHT-based service discovery protocol that constructs topology- aware overlay networks in large-scale WSNs is proposed. In [4], a Chord-based P2P protocol, called Tiered Chord (TChord) that can seamlessly integrate sensor networks with IP networks is described in detail. In [45], CSN, a novel DHT based network protocol for sensor networks is proposed where bounded times for data lookup -in the order of O(logN) messages- may be achieved in an energy efficient manner. CSN follows a hierarchical clustering approach where each cluster is formed in a logical ring. CSN makes system lifetime of the sensor network proportional to its effective use and scales well to large-scale sensor networks.

However, a generic mapping of DHT based protocols to sensor networks is considered challenging for various reasons [45]. These protocols interconnect nodes independently of their physical location and are not able to handle dynamic changes in the sensor network topology. In addition, they prerequisite the maintenance of routing information among all nodes and require unique identifiers for each node. Most of these challenges may be addressed given the existence of an overlay topology formulation and maintenance mechanism and its cooperation with the existing routing and clustering mechanisms.

Several algorithms have been proposed for overlay topology formulation and maintenance that range from gossiping techniques [46] to exhaustive techniques [47]. The common characteristic of these mechanisms is that they presume guaranteed communication among the network nodes. However their principles are diverse. Gossiping techniques attempt to identify the relative position of one node in the overlay topology by consulting adjacent nodes. Alternatively, exhaustive techniques attempt to pass through all nodes periodically in order to identify their relative position in the overlay topology. The topology formulation mechanism has to cooperate with the routing protocol due to the mentioned requisite that any two nodes be able to communicate.

In our approach, as described in detail in Section 4, we propose the exploitation of routing information that is maintained by NEURON via the overlay topology formulation mechanism. In NEURON, routing information is updated continuously in each node’s routing cache and may be consulted from upper layer mechanisms. Given the existence and maintenance of the overlay network, DHT functionality may be applied—using the 6LowPAN address of each sensor as its unique identifier—and advanced services may be provided in an autonomous manner.

## 3. Proposed Protocol

In this section we present the ***N****etwork bas****E****d a****U****tonomic cluste****R****ing and r****O****uti****N****g* ***(NEURON)*** protocol, aiming to address challenges that are accrued from the current trends in the WSNs evolution and the existing approaches for efficient deployment, operation and maintenance of large in scale wireless sensor networks. NEURON is designed in order to achieve the following goals:

- *Support autonomic functionalities in a WSN*: sensors deployed in the sensor field, establish independently communication with their neighbours, become part of the WSN and gain access to the provided data and services without human intervention.
- *Self-organize the nodes into clusters*: clusters are self-formulated according to the current conditions in the network, while cluster heads rotation extends the network lifetime.
- *Achieve fault tolerance and avoid dependence from specific purpose nodes*: clusters are formulated automatically in case of failures, since each node may be elected as CH.
- *Reduce energy consumption through hierarchical routing based on an overlay among the CHs*: information about the current CHs is disseminated among the WSN and routing is provided through them.
- *Achieve scalability by taking advantage of autonomicity, decentralization and probabilistic techniques*: there is no performance degradation as the size of the sensor network increases.

It is important to note that NEURON does not make any assumptions for its operation. It may be applicable to heterogeneous environments, in which nodes with diverse computational and storage capabilities may be present. It does not require prior knowledge about location based information or the pre-assignment of specific roles in the sensor nodes. Its operation is based on the knowledge of network based parameters within the WSN that could be estimated in an autonomous manner by using an *Autonomic Estimation Algorithm*, as described in Section 3.1. NEURON may be effectively applied in static and dynamic topologies where nodes continuously join or leave the network or they are to some extent mobile. Furthermore, its clustering and routing mechanisms—as presented in Sections 3.2 and 3.3—make it energy efficient since they suppress the number of messages that are required for its operation and they support multi-hop communication.

### 3.1. Autonomic Estimation Algorithm

The knowledge of network-based parameters is crucial for achieving high efficiency and optimising mechanisms in a WSN. The estimation of such parameters is challenging in autonomic environments, especially when there is no information available at the initial deployment or after a topology change. For example, in case of clustering, the knowledge of the network density (*i.e*., the average number of single-hop neighbours of each sensor within the WSN) may facilitate the selection of the Hops-To-Live (HTL) parameter for flooding limitation within the WSN. When the density of the network is high the HTL parameter should be small and *vice versa*. Therefore, the knowledge of network-based parameters may impact the efficiency of the mechanisms applied in diverse WSN environments.

NEURON adapts its mechanisms based on network-wide parameters without presuming a priori knowledge of their values. An estimation of the parameters’ values is autonomously produced and updated regularly without imposing significant network overhead in terms of messages exchanged. The mechanism is activated in each sensor when the network bootstraps or the topology changes significantly. It has to converge in a short number of cycles and to be applicable to large scale WSNs. An autonomic mechanism is presented in this paper based on the principles of neighbour-based gossiping and specifically using averaging techniques based on neighbour-based gossiping [36].

In NEURON, each node interacts with its neighbours in order to calculate the mean value of a parameter (Table 1). Each node calculates its initial value for a parameter and sends this value to its neighbours. In parallel each node receives from its neighbours their calculation about this parameter. After each ‘cycle’ of mutual exchanges, each node revises its calculation using a weighting average:

$$\mathrm{UpdatedValue}=\frac{\left(\mathrm{Value}\_\mathrm{from}\_\mathrm{Neighbour}\_1+\cdots +\mathrm{Value}\_\mathrm{from}\_\mathrm{Neighbour}\_n\right)}{n}$$

When the updated value after the completion of a cycle differs less than a specified threshold from the previous one, the parameter is considered as converged on this node. In this case, the node, in the next cycle, sends its converged value along with a flag that indicates that the node considers the parameter-estimation as precise. Only when all messages that are received during a message-exchange-cycle contain this convergence flag, a node decides to stop broadcasting its current value about a parameter. This procedure is repeated periodically in order to calculate the updated values of the network parameters.

This technique presents many advantages since there are no preconditions during the network bootstrapping, the estimation is conducted in an *ad hoc* manner and the algorithm converges quickly, even for large scale networks, while communication overhead is kept low. The frequency of the periodic estimation mechanism is related to the application dynamicity. In NEURON, this technique is used for size and average density estimation. The knowledge of these parameters is indicative for the possible network topology scheme and facilitates the good distribution of cluster heads in the clustering mechanism, as we explain in detail in Section 3.2. However, the same technique may be used for the autonomic calculation of other parameters within the WSN (e.g., variance in the cluster sizes, available energy percentage).

For the density estimation, each sensor node calculates the number of its neighbours and thus the converged parameter is considered to be the average network density. For the size estimation, one or more predefined nodes in the network initialize the parameter *Network_Size* to 1 (at step 0) while the rest nodes initialize the parameter *Network_Size* to 0. When the averaging protocol converges, the estimated value is 1/(*N* · *k*) where *N* is the network size and *k* the number of nodes that initialized the parameter *Network_Size* to 1 [48]. The following adaptation is proposed in NEURON in order to estimate the *Network_Size* autonomically (without the need to predefine specific nodes that initialize the parameter *Network_Size* to 1). All nodes have a random number generator and express their initiative to initialize their *Network_Size* parameter to 1 with a certain probability. We address this probability as *P_init_*. The critical part of the adaptation is that all nodes respect the same probability. *P_init_* varies from 0.1 to 0.3. When the *Network_Size* parameter converges the converged value is approximately 1/(*N · P_init_*). By inversing the converged value, an approximation of the network size is available.

However, this adaptation provides a parameter’s estimation with some variance. In case that we desire to have more precise estimations, each node that chooses to initialize its *Network_Size* parameter to 1 has to accompany the broadcasted message with an additional field called *SolicitatedGroup*. In this field the MAC address of the node is placed. Each node maintains a cache that contains all the solicitated MACs and in parallel, during each exchange of messages, solicits the contents of its cache. Then, in addition to the convergence criteria that were formulated previously, each node is not meant to be converged if the number of MACs that exist in its cache is not equal to the number of MACs that are solicited by its neighbours. Following this adaptation the converged value is exactly (not approximately) [1/(*N* · *NoMacs*)] where *NoMacs* stands for the number of solicited MACs. The advantage of this adaptation is that it generates extremely precise results, albeit at the expense of a larger amount of messages exchanged until convergence is achieved.

### 3.2. Cluster Formulation, Maintenance and Update

The cluster formulation mechanism in NEURON has a significant impact on the WSN from multiple perspectives. It improves the energy efficiency and facilitates the design and deployment of higher layer protocols and applications. Clusters are autonomously formulated, maintained and updated based on neighbour to neighbour communication among the sensor nodes. The *Autonomic Estimation Algorithm* provides information necessary for the optimisation of the CHs selection and distribution. Routing information acquired during the clustering process is stored in nodes’ local caches and used by the routing algorithm applied. Controlled flooding is also utilised in order to avoid traffic forwarding outside the cluster zone.

NEURON allows each node to become a CH according to specific criteria. A node that is selected as CH acts as a proxy for the rest of the members in its cluster. Each node may be in two states; either belonging to a cluster or being in the process of joining to a cluster. The clustering process starts only after the *Autonomic Estimation Algorithm* has been converged and, thus, the size *N* and density *d* of the WSN is estimated. Based on these two parameters, each node decides to become a CH with a probability *P_clust_* given by the following equation:

(1) $$P_{\mathrm{clust}}=\frac{\text{log}(N)}{10*d}*\mathrm{KPI}$$

where:

(2) $$\mathrm{KPI}=0.5*\frac{\mathrm{available}\_\mathrm{battery}}{\mathrm{total}\_\mathrm{battery}}+0.5*\frac{\mathrm{available}\_\mathrm{memory}}{\mathrm{total}\_\mathrm{memory}}$$

The Key Performance Indicator (KPI) in Equation 1 refers to the capabilities of each sensor. Nodes with better KPI present higher probability of becoming CHs and remaining in this status for a longer period of time until their resources are reduced significantly. In our case, the KPI is related with the available battery and memory of each node and is given in Equation 2. These parameters were considered crucial for a sensor node deployment and operation in a WSN. However, any other parameter that better addresses application- specific requirements (for the KPI) may be selected.

According to equation 1, a smaller number of CHs is expected to be elected in dense networks than in sparse ones. In addition, a higher number of CHs is anticipated in larger networks compared to smaller ones. Equation 1 was selected to cover a wide set of possible WSN topologies. However, if the network size or density is known *a priori*, more optimal possibilities *P_clust_* may be selected.

The *P_clust_* is updated regularly based on the current parameters *N* and *d* of the network. This allows NEURON to adapt to changes in the network topology or conditions and optimise the clustering formulation process. For example, in case of a node transitioning from a CH operation mode to normal operation mode, the nodes of the cluster decide to become CHs taking into consideration the latest estimations of the parameters *N* and *d*. In this case, if the average density is reduced, more than one CH may be elected. This process, combined with the cluster formulation update mechanism described in the next paragraph, facilitates the better distribution of CHs among the WSN and the extension in the network lifetime.

#### Cluster Formulation, Maintenance and Update Mechanisms

**Figure 1.**
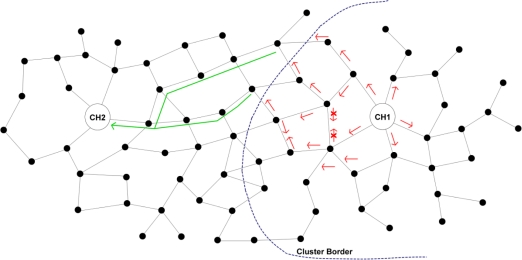
Controlled Flooding Mechanism.

**Figure 2.**
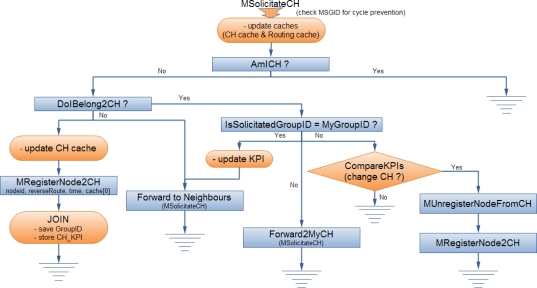
Solicitation Mechanism.

Each CH is responsible for periodically broadcasting its existence utilizing a controlled flooding mechanism. According to this approach, at a certain time interval the CH broadcasts a *MSolicitateCH* message. This message contains its MAC address (which is also the group identifier), its updated KPI and an auto increment number that is used for cycle prevention. The goal for this solicitation message is threefold:

- *Cluster Formulation & Maintenance*: Upon the receipt of a *MSolicitateCH* message by a node not registered to a CH, a *MRegisterNode2CH* response message is send to the CH. The latter updates its routing cache and then the node automatically becomes a member of the broadcasted cluster. If the node belongs to the cluster controlled by the CH generating the message, the node forwards it to its neighbours. In addition, it stores the message to a local cache in order to avoid serving the same message again. Otherwise, if the node does not belong to the cluster controlled by the CH generating the message, the node belongs to the borderline between two clusters, as shown in Figure 1. In this case, the node does not forward the message to its neighbour but instead forwards it directly to its CH. This process is very critical as it prevents the unnecessary flooding out of the scope of a cluster and allows CHs to be aware of their neighbouring CHs. The routing information, collected to the CHs’ caches during the clusters formulation, facilitates the hierarchical routing mechanism, as discussed in Section 2.3. The cluster formulation and maintenance process is also shown in Figure 2.
- *Routing Cache Maintenance:* Each *MSolicitateCH* message—broadcasted by a CH and flooded within the cluster—contains a list of the MAC addresses of the nodes that have already forwarded it since each node that serves this message appends its MAC to this list (only once due to cycle preventive mechanism). This information allows cluster nodes to learn (or update) the shortest path towards their CH and store this information in their local routing cache.
- *Cluster Formulation Update:* The *MSolicitateCH* message allows nodes to be dynamically distributed among the existing clusters according to the CHs KPIs. In case that a node receives a *MSolicitateCH* message from a neighbouring CH, it compares the received KPI with the KPI of its current CH. When this comparison overcomes a specified threshold, the node unregisters from its current CH and registers to the new CH. These tasks are accomplished with the usage of a *MRegisterNode2CH* and *MUnRegisterNodefromCH* message, accordingly. This approach allows CHs to extend their lifetime since the load is re-distributed among the more powerful CHs.

**Figure 3.**
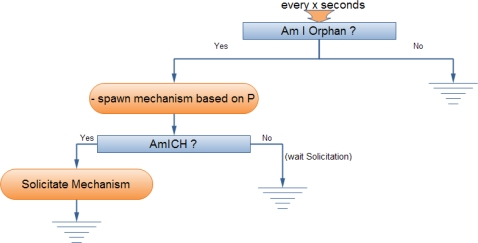
Periodic Mechanism for CH election.

The NEURON clustering mechanism allows the autonomic re-formulation of clusters and enables the adaptation of the clusters’ number and formation to the existing network conditions. This mechanism is initiated either because a CH decides to return to normal operation mode or because the CH leaves the network due to an unforeseen situation. In the first case, when the KPI of the CH passes below a specified threshold, the node stops to undertake the role of CH and a *MSolicitateCHDOWN* message is flooded within its cluster. Nodes update their routing cache and inform neighbouring CHs, provided that routing information for them is available in their routing cache. The re-clustering mechanism is then invoked and the cluster members decide to become a CH with the current probability *P_clust_*. If no CH is elected, the nodes join a neighbouring cluster after receiving a neighbouring *MSolicitateCH* message. In the second case, if the *MSolicitateCH* message is not received within a period, the node updates the routing cache and initiates the re-clustering mechanism as previously (Figure 3). It should be noted that the CHs remove any entries from their routing cache related with neighbouring CHs if no relevant *MSolicitateCH* message is received within a specific period.

### 3.3. Hierarchical Routing

Routing and clustering mechanisms are interrelated in WSNs, both of them targeting to minimise energy consumption. It is desirable that packet forwarding and routing protocol overhead is distributed among all the sensor nodes according to their KPI values. This approach preserves scarce sensors recourses and, thus, extends the network lifetime.

Energy efficiency in NEURON is achieved by hierarchical reactive routing. Nodes are organized into a hierarchy of clusters based on network proximity to the CHs. There is no proactive mechanism to build and maintain a valid routing table as the network topology continuously evolves. In addition, routing mechanism takes advantage of the routing cache entries generated during the clustering process, as presented in Section 3.2.

The routing algorithm in NEURON presents similarities with the DSR routing protocol [40]. NEURON adopts some mechanisms from DSR for communication among CHs, while intra-cluster communication is designed independently. A *RouteRequest* message is used for detecting a valid route to a destination node, in accordance to the DSR protocol. In NEURON, however, the *RouteRequest* message is not flooded but directly forwarded to the CH of the transmitting node. The exact path to the CH is known via the *MSolicitateCH* messages broadcasted by CHs in regular intervals.

**Figure 4.**
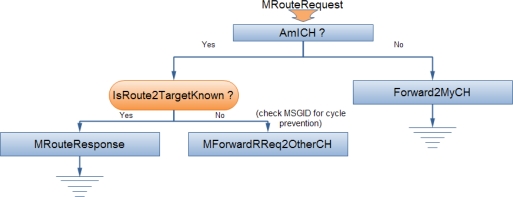
Routing in NEURON.

**Figure 5.**
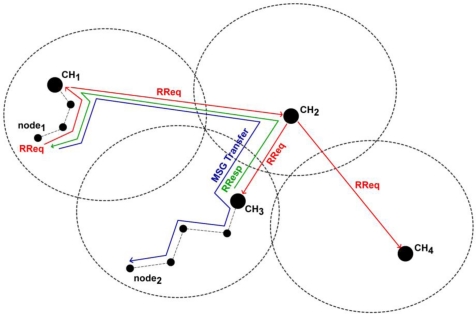
Route Request Mechanism.

When a node desires to establish communication with another node, it initially sends a *RouteRequest* message to its CH (Figure 4). The message contains the exact path towards the CH (source routing). If a node receives a *RouteRequest* message, it initially checks whether it operates as a CH. This is necessary because the election of new CHs is a dynamic process and thus new CHs may be present. If the node is not a CH, then it forwards the message to the next hop towards the CH according to the disseminated path from the initiator node. In case that a node along a path is unreachable, a *RouteError* message is generated and broadcasted within the scope of the cluster that contains the broken link. If the message is delivered to a CH during its path, the CH queries its local cache for the requested route towards the destination. If the valid entry is found, a *RouteResponse* message is sent to the initiating node. Otherwise, the CH forwards the *RouteRequest* message to its known CHs, exactly as routing is implemented in DSR. The CH of the destination node will directly reply to the initiator node with the correct end-to-end path (Figure 5).

When a route to a destination is known then a *Message_Transfer* message is initiated. This message contains the source node, the destination node, the route that must be followed in the WSN in order to reach the destination and a flag that informs the destination node whether it should respond with a confirmation (acknowledgement). The *Message_Transfer* message is used for transferring upper layer data, e.g., overlay topology formulation.

### 3.4. Requirements from Sensor Node Platforms

No special requirements are imposed by NEURON in order to be applied to existing and next generation sensor motes. In NEURON, nodes may be identified according to their 6LoWPAN address while low power 802.15.4 radio may be used for communication among them. According to 6LowPAN, IEEE 802.15.4 devices may use either IEEE 64 bit extended addresses or 16 bit addresses that are unique within a Personal Area Network (PAN).

NEURON, as stated earlier, necessitates the storage of routing information in the routing cache of each node. This information regards the routes that are stored in each node during the cluster formulation and maintenance process. Considering that the maximum depth (distance in hops) from a CH is approximately five, the size of each routing entry in NEURON varies from 10 to 15 bytes (when nodes are indentified by their 16 bit addresses) or from 20 to 45 bytes (when nodes are indentified by their IEEE 64 bit extended addresses). Thus, in case of a routing cache with 1,000 units, the maximum memory that could be necessary is 45 kB (in case that all the routes are more than four hops) while in a realistic scenario 15 kB are sufficient, as also shown in Section 5.2.

Requirements for energy depend on the type of the sensor mote. However, the behaviour of NEURON regarding energy efficiency is presented in detail in Section 5. Finally, NEURON does not impose special processing requirements or constraints for the operating system used, since NEURON mechanisms perform simple functionalities that may be implemented in each operating system.

## 4. Service Provisioning Over NEURON

The creation of an overlay topology is beneficial for decentralized and autonomic service provisioning in WSNs. Multiple challenges, though, have been identified due to the dynamic network characteristics in such networks. P2p protocols may efficiently manage the sensors’ interconnections (as nodes continuously join or leave from the overlay network) or control the autonomic delegation of tasks among participating nodes [10,42]. Addressing scalability and complexity challenges, though, is still a research issue [49]. NEURON exhibits the necessary functionality to upper layer protocols in order to establish an overlay network while aims to address complexity and scalability issues. It also provides routing information to topology formulation mechanisms in order to improve their efficiency.

Several topology formulation algorithms have been proposed in the literature [41,46,47]. The T-MAN algorithm was selected for investigating the advantages that NEURON may offer to the overlay topology formulation mechanisms due to the faster convergence capabilities of T-MAN compared to other alternatives for the creation of an overlay topology [50]. T-MAN allows communications with any node in the overlay network contrary to other gossiping protocols that permit communications with only the one-hop-away neighbours (refer to Section 3.1). The latter approach increases routing overhead as one node has to identify a proper route prior to attempt to communicate with another node in the overlay network. In T-MAN, each node aims to identify its successor in the overlay topology based on the knowledge that acquires through the exchange of views with its neighbours. Scoring functions are applied for the selection of the successor of each node and the results are stored in a buffer. The scoring function affects the formation of the overlay network topology. In our case, a ring topology is formulated since Chord [42] was selected as a p2p protocol for the provision of storage and retrieval functionality over the created overlay network. Chord pre-assumes that nodes are ordered in a ring and are aware of their successor and predecessor in the overlay ring topology. However, any other topology may be also created based on the selected p2p protocol.

**Figure 6.**
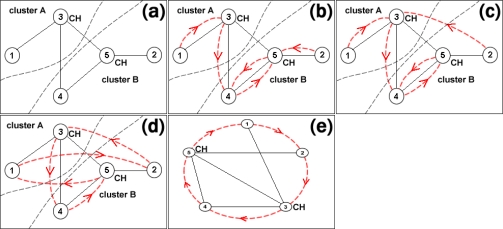
Topology Formulation Algorithm.

T-MAN applies a gossiping technique [50] in order to indentify the relative position in the overlay (Table 2). Each node maintains a view with the nodes that are—up to a specific time—known and scored. Each node periodically communicates with the “closest” node and exchange views with it (Figure 6a,b). After this mutual exchange, nodes re-evaluate their views (Figure 6c,d). This iterative procedure leads to extremely fast convergence, *i.e*., the state in which each node knows its successor. Any further exchange of messages between the nodes does not improve the accuracy of their views (Figure 6e).

**Figure 7.**
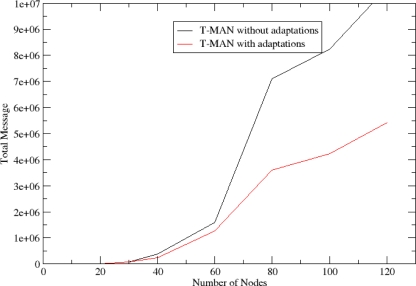
Total messages for overlay topology formulation.

The T-MAN algorithm is adapted in order to exploit information available through the NEURON mechanisms and thus minimize the messages that are necessary for convergence of the protocol in a WSN. Two improvements have been integrated to the T-MAN algorithm. Firstly, each node, before sending its buffer to a requestor, updates the buffer with nodes that score better than the existing ones by consulting its routing cache. Secondly, multiple messages are sent to any node in the buffer instead of sending one message to the first node of the buffer (i.e. the node that scores better). This facilitates the fast dissemination of information regarding the network topology which is critical in dynamic networks. These two adaptations reduce the total amount of messages required for overlay topology formulation, as shown in Figure 7.

After the overlay network is established, participating nodes are able to store and retrieve data using typical p2p protocols. Every node that aims to access the p2p network storage (*i.e*., to store a key/value pair or query a value based on a key) may use a Distributed Hash Table (DHT) [51] that operates on-top of the overlay topology. In our simulation experiments, Chord [42] was selected for integrating DHT functionality. Chord is an efficient distributed lookup system based on consistent hashing. Its only operation is to map a key to a responsible node. Chord scales well with a number of nodes and, thus, it can be applicable to large networks. It continues to function correctly even if the system undergoes major changes or if the routing information is partially correct [42].

**Figure 8.**
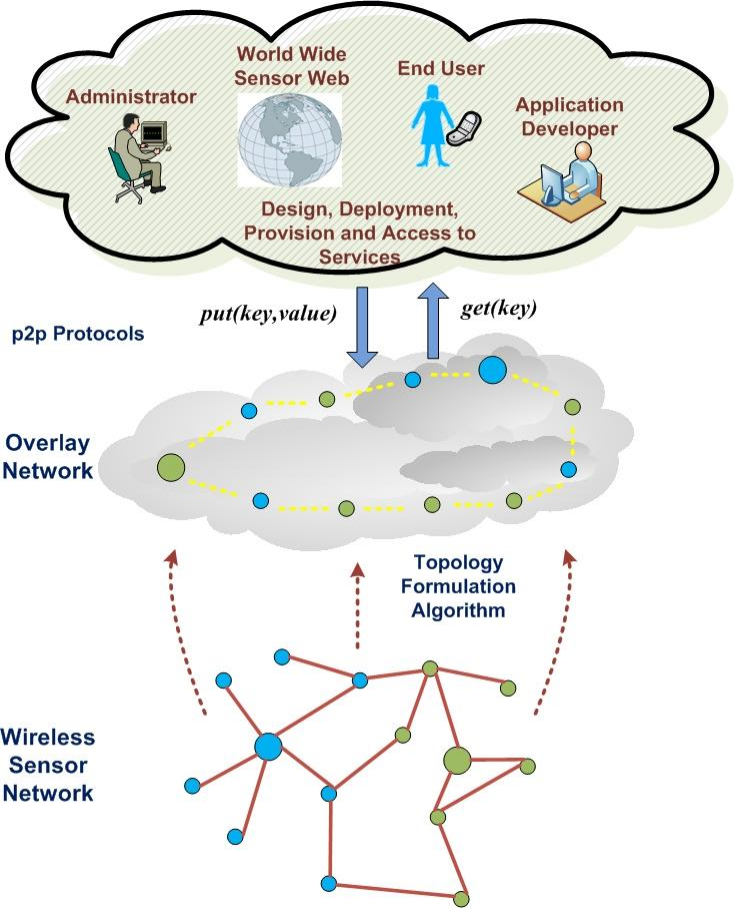
Autonomic Provision of Services.

Advanced services may be built using two API functions *put(key,value)* and *get(key),* which interact directly with the DHT protocol (Figure 8). For instance, a distributed storage system handling environmental monitoring data may be built by using common hashing functions and predefined keys [42]. Environmental data is consequently available for retrieval and further processing by all network nodes. The provided services could be decentralized as data and necessary functionality is allocated in multiple nodes at the overlay network. If necessary, some critical functions may be delegated to more than one nodes for improving reliability. In case of network changes or node failures, roles may be re-assigned autonomously and performance guarantees may be assured for the services provision.

## 5. Experimental Evaluation

In this section the performance of NEURON for a wide set of topologies is evaluated. NEURON has been developed in the Peersim simulator [52]. A visualisation module is also developed as a Peersim extension that provides a view of the clusters with their CHs that are formulated in the WSN at each cycle period of the simulation. In order to simulate the limited resources of the participant nodes of the WSN, a custom dynamic model is incorporated that imposes penalties according to the nodes operations.

In the simulations multiple nodes are simultaneously activated without any preconfigured state information. Each simulation lasts 2,000 cycles, while every node is initialized with 100,000 battery units and 1,000 routing cache memory units. Battery and memory penalties are defined for serving each message in the network. Each entry in the routing cache occupies one memory unit, each packet transmission or reception drains the available battery by three units, while each packet processing action (e.g., protocol encapsulation) that is accomplished by a node drains the battery by one unit. The periodic broadcasting of *MSolicitateCH* messages is set to five cycles, the KPI threshold (refer to Section 3.2) for transition to a new CH is set to two, and the threshold where a CH switches to normal mode (refer to Section 3.2) is set to 50%. All the nodes are considered with equal battery and memory capabilities at their initial deployment. The number of nodes varies from 50 to 12,800 while the density varies from three to 36.

The performance of each mechanism is assessed using multiple criteria, such as messages exchanged for the operation in steady state, convergence capability, precision in the estimation of parameters, behaviour of the probabilistic techniques, energy efficiency and quality of distribution of CHs in diverse network sizes and densities. Simulation results that are related with the creation of the overlay network and NEURON’s suitability for deployment of advanced services in the WSN are also presented. Each simulation is executed five times and average values are considered in our analysis.

### 5.1. Evaluation of the Autonomic Estimation Algorithm

The *Autonomic Estimation Algorithm,* aiming to estimate two network parameters, is activated right after the sensor nodes become operational. In each node, the algorithm converges if the estimated parameters’ values between two consecutive cycles is less than 5%.

**Figure 9.**
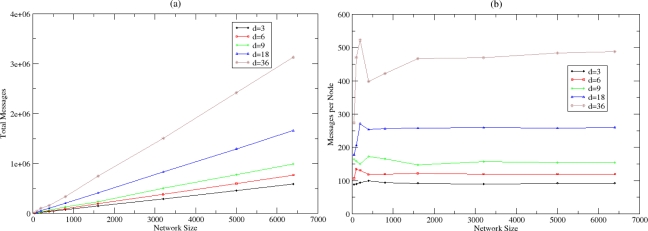
(a) Messages for Network Density and Size Estimation and (b) Messages for Network Density and Size Estimation per node.

Figure 9(a) shows the number of messages that are exchanged until the algorithm converges to the estimation of the density and the size parameter for various sizes and densities. Figure 9(b) shows how these messages are distributed between nodes. There is a linear relationship between the total number of messages exchanged and the network size. It derives that the algorithm convergences without imposing significant overhead since the average number of messages per node remains small and stable even for large-scale networks. The autonomic estimation process may be repeated periodically in predefined number of cycles, related with the dynamicity that is present in the WSN.

**Figure 10.**
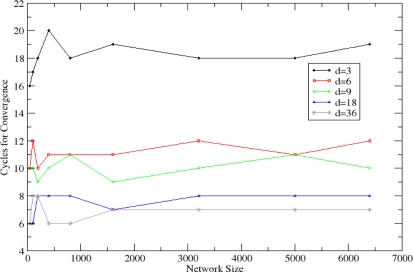
Cycles for convergence of the estimated parameters.

Figure 10 presents the cycles that are necessary for the algorithm to converge. Stable behaviour is achieved for each network density independently from the network size. The algorithm converges in less than 20 cycles in sparse networks and in less than 10 cycles in dense networks, independently of the network size. In dense networks, convergence is faster since more messages are exchanged at each cycle.

**Figure 11.**
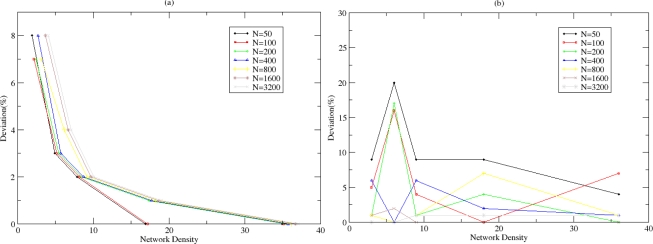
Deviation in the estimation of the (a) density and (b) size of the WSN.

The *Autonomic Estimation Algorithm* achieves adequate precision for the estimation of the network parameters, as presented in Figure 11(a) for the density estimation and Figure 11(b) for the size estimation. For the density estimation, the deviation from the real values is less than 9% in all cases and remains approximately constant for a given network density. For the size estimation, the deviation is less than 20% for sparse networks and less than 10% in dense networks. In both cases, higher precision is noticed in dense networks since averaging is performed between multiple neighbours in each cycle.

### 5.2. Clustering and Routing Mechanism Evaluation

**Figure 12.**
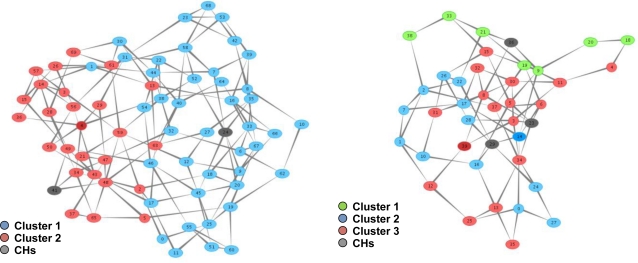
Cluster Visualisation.

A visualisation module is developed for the dynamic illustration of the clustering process and the distribution of the CHs within the WSN. It is noticed that the clusters’ distribution improves (qualitative metric) over time since the probabilistic techniques used tend to homogenize the size and form of the created clusters and thus distribute the clustering overhead among the elected CHs. Two indicative screenshots are presented in Figure 12 where clusters are distinguished.

**Figure 13.**
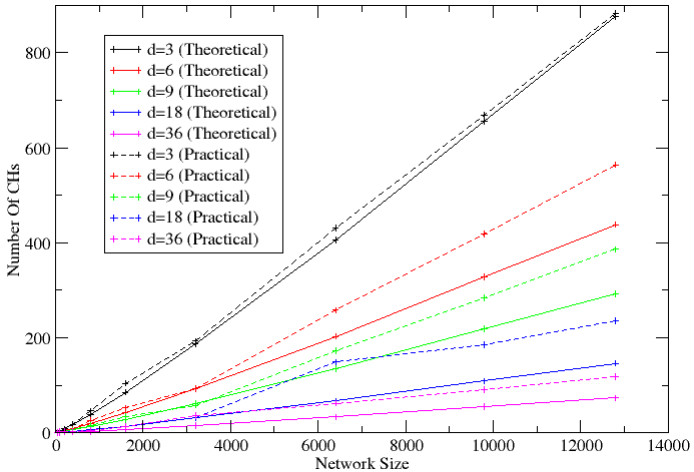
Theoretical and Practical number of elected CHs.

In Figure 13, the comparison of the number of CHs that are elected in the simulation environment with the theoretical ones according to the *P_clust_* (refer to Equation 1) is shown. As it is expected, the theoretical and simulation results are closely related.

**Figure 14.**
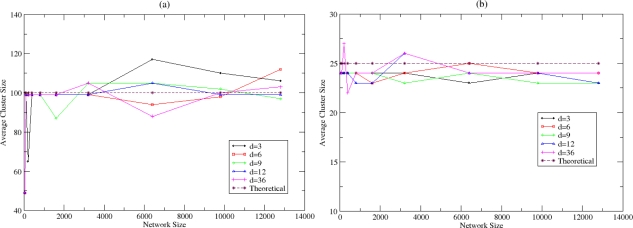

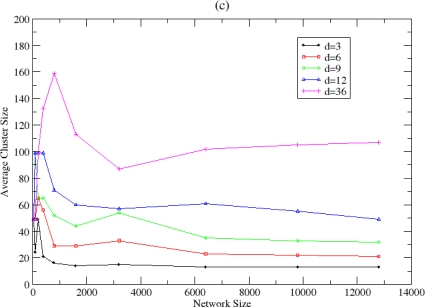
Cluster Size for (a) *P_clust_* = 1%, (b) *P_clust_* = 4% and (c) variable *P_clust_*.

An important characteristic for the optimisation of the clustering process is the adaptation of the clusters’ size according to the changes in the network topology. In cases of more dense networks, it is desirable the creation of larger (in size) clusters since nodes are close (in number of hops) to each other. The existence of less CHs with small distances from their members improves the energy efficiency of the WSN (refer also to Section 5.3). In Figures 14(a)–(c), the average size of the clusters that are created is presented for fixed and variable probability (refer to Equation 1). In the latter case, the trend is the creation of larger in size clusters in dense networks in opposition to the first case where the cluster size remains stable. Self-optimisation of the clustering process is therefore achieved. However, more optimal equation for the *P_clust_* probability may be selected, in case that smaller clusters are desirable in dense networks. Furthermore, great variation is present in small-scale networks (Figure 14(c)) due to the impact that has the probability in the cluster size, as the number of the elected CHs significantly affects the average cluster size. This variation is decreased as the period that NEURON is applied in the WSN increases since probabilistic techniques follow an optimal behaviour.

**Figure 15.**
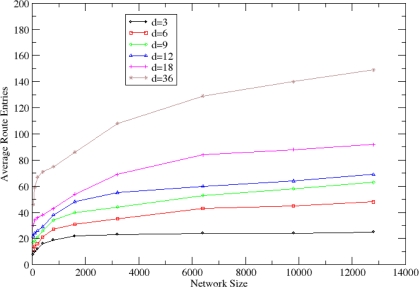
Average entries in each node’s routing cache.

The average number of route entries that are stored on each node’s routing cache after the cluster formulation process is shown in Figure 15. This number is critical since sensor nodes may present memory constraints. *P_clust_* is variable according to Equation 1. It is shown that the number of route entries increases slightly as the size and the density of the network increases. However, this number remains bounded, even for large networks. In sparse networks, an average routing cache has 50 entries, while in dense networks an average routing cache with 150 entries is needed. Since the maximum size of a routing entry is 45 bytes (refer to Section 3.4), 15kB of routing cache size is adequate for all the sensor nodes.

**Figure 16.**
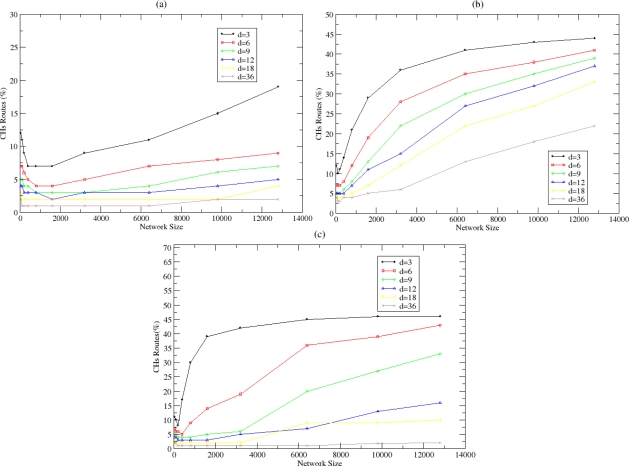
Percentage of total routes in the CHs routing caches for (a) *P_clust_* = 1%, (b) *P_clust_* = 4% and (c) variable *P_clust_*.

In addition to the number of route entries, a qualitative metric is the percentage of the total route entries that exists in the CH’s routing caches since these entries are used from the routing functionality in NEURON. This metric is depicted in Figures 16(a)–(c) for fixed and variable probability. As the number of CHs increases in the network, the percentage of the total routing entries that exist in their routing caches also increases. This is shown in Figures 16(a,b), where the selection of larger value for the stable probability results to higher percentages of routing cache entries in the CHs. When this percentage is smaller, intra-cluster communication is facilitated since the nodes that are not CHs have available routes for other nodes within the cluster and do not need to communicate with their CH for establishing a route towards them. This percentage is smaller for dense networks due to the greater overlapping among the cluster zones and the existence of multiple paths toward a CH. Nodes in the overlapping regions store routing entries towards more than one CH. The percentage also increases as the size of the network increases while when a variable probability is used (Figure 16(c)), the percentage is small for dense networks and large for sparse networks.

**Figure 17.**
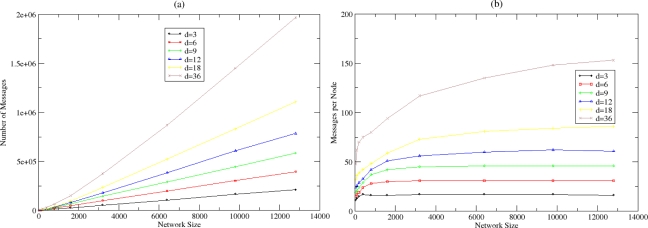
(a) Total number of messages exchanged for cluster formulation and (b) total number of messages exchanged for cluster formulation per node.

In Figure 17(a), the total number of routing messages that are exchanged until the clustering formulation is completed is presented, while in Figure 17(b), the same number per node in the WSN is shown. More routing messages are exchanged in dense networks, due to the nature of the controlled flooding mechanism that has been adopted from the *MSolicitateCH* message. Although cluster formulation messages are confined and cycle-prevented as discussed earlier, the existence of multiple connections for each node creates an analogous routing overhead that is avoided in sparse networks. Furthermore, in Figure 17(b) it is shown that the clustering formulation mechanism is scalable since the number of messages per node for different densities remain either stable or slightly increases as the network size increases.

**Figure 18.**
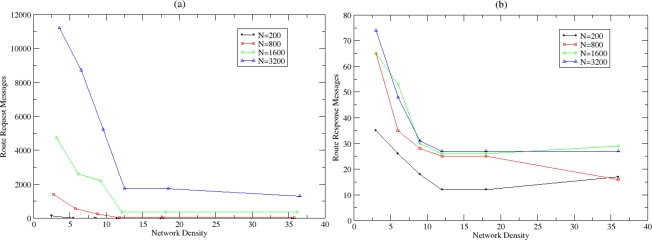
(a) Route Request and (b) Route Response Message Cost.

Upon completion of the clustering process, routing functionality exploits the available information in the routing caches of the sensor nodes. In Figures 18(a) and 18(b) the total number of *RouteRequest* and *RouteResponse* messages that are exchanged in order a node to identify a valid route, are depicted. The number of the generated *RouteRequest* and *RouteResponse* messages is radically reduced as the network density increases since more routing information is already available in the nodes. Thus, NEURON’s scalability is addressed as the routing overhead (in number of messages) is considered low.

### 5.3. Energy Efficiency in NEURON

Simulations are performed in order to assess the energy efficiency of NEURON mechanisms and their impact to the network lifetime. Network lifetime refers to the time period where all the nodes of the network (or a very high percentage of them) are operational. The network size is set to 1,000 while the initial energy of each node is set to 100,000 units. Simulations are terminated when the existing WSN is split into two or more isolated groups as nodes leave the WSN when their battery is exhausted.

**Figure 19.**
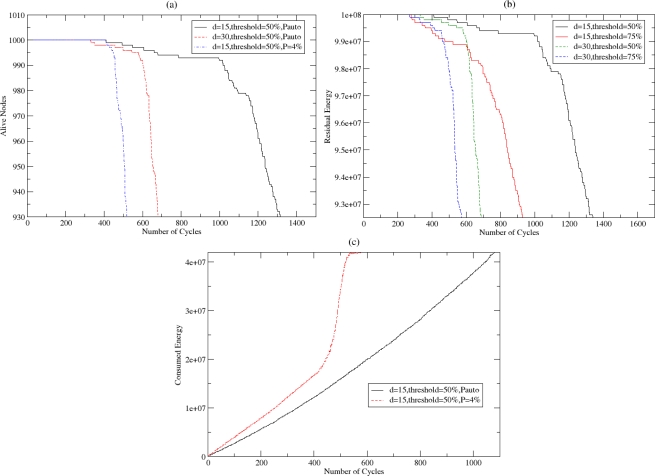
(a) Alive Nodes in the WSN, (b) Residual Energy in the WSN and (c) Consumed Energy in the WSN.

In Figure 19(a), the number of nodes that are alive while the number of cycles increases is shown for fixed and variable probability and different densities. The threshold where a CH switches to normal mode—when the KPI of the CH goes below it—is set to 50%. It is noticed that the network tends to extend its network lifetime as the number of alive nodes reduces steeply after a certain number of cycles. This means that the available power of the sensor nodes is almost the same and thus they run out of power in a few cycles. Furthermore, it is shown that the network lifetime is longer in the case of applying the variable probability and in case of more sparse networks. This is reasonable since fewer messages have to be exchanged in sparse networks for cluster formulation and maintenance. In Figure 19(b), the residual energy in the network is shown. The threshold where a CH switches to normal mode is set to 50% and 75% while the density is set to 15 and 30, respectively. The residual energy is higher in case of sparse networks and in the case where the threshold is set to 50%. A high threshold reduces the rotation in the CHs in the WSN and causes high energy consumption in each CH, causing them to run out of energy earlier than the other nodes. In this case, therefore, energy consumption is not homogeneously distributed among the sensor nodes. In Figure 19(c), the consumed energy is depicted in case of the fixed and the variable probability, while the density is set to 15 and the threshold where a CH transits to normal mode is set to 50%. It is clear that the application of the autonomic mechanism in the election of clusters is more energy efficient compared to the application of a stable probability.

### 5.4. Topology Formulation Mechanism Evaluation

**Figure 20.**
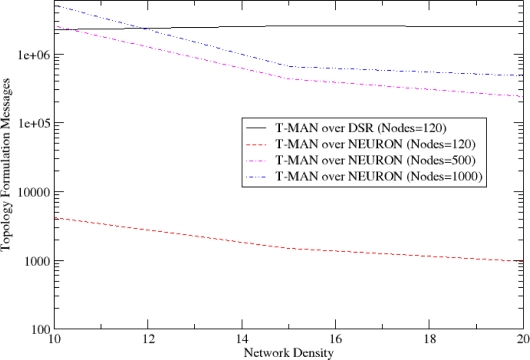
Routing Message Cost for Topology Formulation (logarithmic scale).

As described in Section 4, the NEURON protocol facilitates the creation of an overlay topology and consequently the deployment and provision of autonomic services over it. In order to show NEURON’s suitability for this purpose, we compare the messages that are generated for the overlay topology formulation using DSR [40], *i.e.*, another reactive routing protocol. In Figure 20, it is shown that the logarithmic behaviour of routing cost in DSR imposes extreme overhead to the network in comparison to NEURON. Furthermore, this overhead is much greater in dense networks.

## 6. Conclusions

NEURON, an innovative protocol for autonomic clustering and routing in wireless sensor networks, is presented in this paper. Self-configuration and self-optimisation properties are supported by the proposed set of mechanisms. The use of probabilistic techniques combined with decentralized approaches and cooperation among nodes for dissemination of useful information provide reliability, robustness, energy efficiency and scalability in NEURON’s mechanisms. In addition to the deployment of autonomic functionalities within the WSN, NEURON facilitates the creation of overlay topologies over the WSN, without imposing significant overhead. Overlay networks may be proven extremely useful for the development of advanced services in the sensor networking world, taking into consideration the vision for a World-Wide Sensor Web.

The behaviour of the protocol is evaluated according to a wide set of simulations. It could be argued that NEURON behaves well for autonomic setup and maintenance of clusters while information from network-based estimation techniques may be used for optimisation purposes. Routing information collected during the clustering process proves to be valuable since the number of messages that have to be exchanged for communication among the sensor nodes is reduced. NEURON does not impose severe requirements for memory usage in the sensor nodes and achieves significant extension to the network lifetime through the rotation of CHs in the WSN field.

In our future work, each of the fundamental mechanisms of the NEURON protocol will be compared with other existing protocol’s mechanisms. The behaviour of the described probabilistic techniques will be studied in detail, and possible optimisations may be proposed for diverse network topologies. Furthermore, the performance of some indicative services provided over the overlay network will be examined. Finally, the efficiency of mechanisms that create topology-aware overlay networks over NEURON will be studied.

## Figures and Tables

**Table 1.** Averaging through Neighbour-gossiping.

converged=false; neighbconverged=false; do forever{ if (cycle mod resetcycle ==0){ converged=false; neighbconverged=false; paramvalue = CountNeighbors(); ReceivedMSG=0; } if (ReceivedMSG!=0){ oldparamvalue= paramvalue; paramvalue=(paramvalue) / ReceivedMSG; if (Abs (oldparamvalue- paamvalue) <threshold){ converged=true; } } if (neighbconverged==false){ for (i=0;i<Neighbors.size();i++){ Send Frame[paramvalue,coverged] to Node_i_ } neighbconverged=true; } }	On_Message_Receive_Event{ paramvalue+=ReceivedFrame [paramvalue]; if (ReceivedFrame [converged]==false) neighbconverged=false; ReceivedMSG++; }

active thread	passive thread

**Table 2.** T-MAN pseudo code.

do forever{ Node_To_Sent_p_ ← selectCloserNode() buffer ← merge (view,{myNodeDescriptor}) send buffer to Node_To_Sent_p_ receive buffer_p_ from Node_To_Sent_p_ buffer ← merge (buffer_p_ ,view) view ← Reevaluate(buffer) }	do forever{ receive buffer_q_ from Sender_q_ buffer ← merge (view,{myNodeDescriptor}) send buffer to Sender_q_ buffer ← merge (buffer_q_ ,view) view ← Reevaluate (buffer) }

active thread	passive thread
